# Genome-wide association study in minority children with asthma implicates *DNAH5* in bronchodilator responsiveness

**DOI:** 10.1038/s41598-022-16488-6

**Published:** 2022-07-22

**Authors:** Jaehyun Joo, Angel C. Y. Mak, Shujie Xiao, Patrick M. Sleiman, Donglei Hu, Scott Huntsman, Celeste Eng, Mengyuan Kan, Avantika R. Diwakar, Jessica A. Lasky-Su, Scott T. Weiss, Joanne E. Sordillo, Ann C. Wu, Michelle Cloutier, Glorisa Canino, Erick Forno, Juan C. Celedón, Max A. Seibold, Hakon Hakonarson, L. Keoki Williams, Esteban G. Burchard, Blanca E. Himes

**Affiliations:** 1 Department of Biostatistics, Epidemiology and Informatics, University of Pennsylvania, 402 Blockley Hall, 423 Guardian Drive, Philadelphia, PA 19104 USA; 2 Department of Medicine, University of California, San Francisco, UCSF, 1550 4th Street, Bldg 19B, San Francisco, CA 94158 USA; 3 Center for Individualized and Genomic Medicine Research, Department of Internal Medicine, Henry Ford Health System, Detroit, MI USA; 4 Center for Applied Genomics, Children’s Hospital of Philadelphia, Philadelphia, PA USA; 5 Division of Human Genetics, Department of Pediatrics, The Perelman School of Medicine, University of Pennsylvania, Philadelphia, PA USA; 6 Department of Medicine, Channing Division of Network Medicine, Brigham and Women’s Hospital and Harvard Medical School, Boston, MA USA; 7 PRecisiOn Medicine Translational Research (PROMoTeR) Center, Department of Population Medicine, Harvard Medical School and Harvard Pilgrim Health Care Institute, Boston, MA USA; 8 Department of Pediatrics, University of Connecticut, Farmington, CT USA; 9 Behavioral Sciences Research Institute, University of Puerto Rico, San Juan, PR USA; 10 Division of Pediatric Pulmonary Medicine, UMPC Children’s Hospital of Pittsburgh, University of Pittsburgh, Pittsburgh, PA USA; 11 Center for Genes, Environment and Health, National Jewish Health, Denver, CO USA; 12 Department of Bioengineering and Therapeutic Sciences, University of Californica, San Francisco, CA USA

**Keywords:** Genetic association study, Asthma

## Abstract

Variability in response to short-acting β_2_-agonists (e.g., albuterol) among patients with asthma from diverse racial/ethnic groups may contribute to asthma disparities. We sought to identify genetic variants associated with bronchodilator response (BDR) to identify potential mechanisms of drug response and risk factors for worse asthma outcomes. Genome-wide association studies of bronchodilator response (BDR) were performed using TOPMed Whole Genome Sequencing data of the Asthma Translational Genomic Collaboration (ATGC), which corresponded to 1136 Puerto Rican, 656 Mexican and 4337 African American patients with asthma. With the population-specific GWAS results, a trans-ethnic meta-analysis was performed to identify BDR-associated variants shared across the three populations. Replication analysis was carried out in three pediatric asthma cohorts, including CAMP (Childhood Asthma Management Program; n = 560), GACRS (Genetics of Asthma in Costa Rica Study; n = 967) and HPR (Hartford-Puerto Rico; n = 417). A genome-wide significant locus (rs35661809; *P* = 3.61 × 10^–8^) in *LINC02220*, a non-coding RNA gene, was identified in Puerto Ricans. While this region was devoid of protein-coding genes, capture Hi-C data showed a distal interaction with the promoter of the *DNAH5* gene in lung tissue. In replication analysis, the GACRS cohort yielded a nominal association (1-tailed *P* < 0.05). No genetic variant was associated with BDR at the genome-wide significant threshold in Mexicans and African Americans. Our findings help inform genetic underpinnings of BDR for understudied minority patients with asthma, but the limited availability of genetic data for racial/ethnic minority children with asthma remains a paramount challenge.

## Introduction

Asthma, a common chronic respiratory disease among children, is a serious global health burden^1^. Inhaled short-acting β_2_-agonists (SABAs), such as albuterol, have been the first-line bronchodilator therapy for acute treatment of asthma symptoms. SABAs act on β_2_-adrenergic receptors to cause a prompt relaxation of bronchial smooth muscle cells (i.e., bronchodilatation) via generation of intercellular cAMP and subsequent activation of cAMP-dependent protein kinase A (PKA)^2^. Despite their widespread use, however, patients’ responses to SABAs are highly variable^3^. Identifying the sources of this variability among patients is important to tailor preventive and treatment strategies.

Bronchodilator response (BDR), measured as the percent change in forced expiratory volume in 1 s (FEV_1_) after inhalation of a SABA, is an important phenotype used for asthma diagnosis, assessing its severity, and determining whether symptoms are under control. There has been a growing recognition that inter-individual variability in BDR is due in part to genetic factors^4^. Previous studies have shown that BDR in people with asthma substantially varies by race/ethnicity^5–7^. In patients with moderate-to-severe persistent asthma, BDR was lower in African Americans compared to whites^5^. Among Hispanic groups, Puerto Ricans were less responsive to albuterol than Mexicans, had decreased lung function, greater morbidity, and longer asthma duration^6,7^. Genetic association studies have reported several BDR-associated genes, including *ADRB2*^8,9^, *ADCY9*^10^, *ADCYAPR1*^11^, *ARG1*^12^, *COL22A1*^13^, *CRHR2*^14^, *GLCCI1*^15^, *GSNOR*^16^, *THRB*^17^, and *SPATS2L*^18^. However, to date, most of these genetic studies were performed in populations of European ancestry. There are likely other genes that contribute to BDR variability that can be identified in non-European populations of diverse genetic ancestry. Indeed, there is evidence of population-specific variants associated with BDR^19,20^, underscoring the need for additional research among underrepresented racial/ethnic minority groups. A recent genome-wide association study (GWAS) among minority children with asthma using the tails of high and low BDR as outcome found population-specific and shared risk variants, although replication of primary findings was limited due to lack of additional independent cohorts representing the same minority populations^4^. In this study, we conducted the largest BDR GWAS to date using as outcome its continuous distribution.

## Methods

### Study cohorts and sample details

SAGE II (Study of African Americans, Asthma, Genes, and Environments) and GALA II (Genes-environments and Admixture in Latino Americans) are parallel studies that aim to investigate gene-environmental interactions in asthma focusing on African American and Latino children, respectively^21^. Participants were eligible if they were 8–21 years old at time of recruitment. Parents and grandparents must have self-identified as African American (SAGE II) or Latino (GALA II). Exclusion criteria included the following: (1) 10 or more pack-years of smoking; (2) any smoking within one year of recruitment date; (3) pregnancy in the third trimester; or (4) history of lung diseases other than asthma or chronic illness. Asthma status was confirmed with a history of physician-diagnosed asthma and asthma controller or rescue medication use, and report of symptoms within the last two years preceding enrollment.

Additional African American patients with asthma were drawn from the SAPPHIRE (Study of Asthma Phenotypes and Pharmacogenomic Interactions by Race-Ethnicity)^22^ and GCPD-A (Genetic Causes of Complex Pediatric Disorders—Asthma)^23^ studies. SAPPHIRE subjects met the following criteria: (1) self-identified as African American; (2) age 12–56 years; (3) a prior clinical diagnosis of asthma; (4) no record of chronic obstructive pulmonary disease or congestive heart failure. GCPD-A subjects consisted of children and young adults aged 5–21 years seen and followed in the outpatient clinics of the Children’s Hospital of Philadelphia (CHOP). These patients have detailed longitudinal clinical information which includes records of clinical diagnoses. Asthma cases had a documented diagnosis of asthma in the medical record, whereas GCPD-A controls had no documented asthma diagnosis. GCPD-A participants with asthma who completed pulmonary function tests were included in this study.

Replication analyses were conducted in 560 white participants from the CAMP (Childhood Asthma Management Program) clinical trial, in 967 participants from the GACRS (Genetics of Asthma in Costa Rica), and in 417 participants from the HPR (Hartford-Puerto Rico) study. CAMP was a randomized, placebo-controlled trial of budesonide, nedocromil, or placebo for mild-to-moderate childhood asthma followed by three phases of observational follow-up^24^, where asthma was defined by having 2 or more symptoms per week, using an inhaled bronchodilator at least twice weekly or asthma medication daily, and airway responsiveness to methacholine < 12.5 mg/ml. BDR assessed at randomization in the CAMP clinical trial was used in this study. GACRS was a family-based genetics study of childhood asthma comprised of Costa Rican schoolchildren with asthma ages 6 to 14 years and their parents^25,26^. Children had a high probability of having at least six great-grandparents born in the Central Valley of Costa Rica and were defined as having asthma if they had a doctor’s diagnosis of asthma and at least two respiratory symptoms or asthma attacks in the year prior to enrollment in the study^25,26^. HPR was a case–control study of childhood asthma in Puerto Ricans recruited in Hartford, CT and San Juan, PR with asthma defined as physician-diagnosed asthma and at least one episode of wheeze in the year prior to recruitment^27^. All participants had to have four Puerto Rican grandparents.

### Ethics approval and consent to participate

All Human Subjects data utilized was de-identified and previously collected via studies in which participants and/or their parents provided written informed consent. The SAGE II and GALA II studies were approved by the University of California, San Francisco Human Research Protection Program Committee; the SAPPHIRE study was approved by the Institutional Review Board of Henry Ford Health System; the GCPD-A study was approved by the Institutional Review Board of the Children’s Hospital of Philadelphia. The CAMP study was approved by the Institutional Review Boards of Brigham and Women’s Hospital and the other participating centers. The GACRS study was approved by the Institutional Review Boards of the Hospital Nacional de Niños (San José, Costa Rica) and Brigham and Women’s Hospital. The HPR study was approved by the Institutional Review Boards of the University of Puerto Rico (San Juan, PR), Brigham and Women’s Hospital, and the University of Pittsburgh. All research was performed in accordance with relevant guidelines and regulations.

### BDR assessments

BDR was evaluated according to American Thoracic Society/European Respiratory Society Guidelines^28^. Briefly, FEV_1_ was measured before administration of albuterol (pre-FEV_1_) and again after two doses of albuterol (post-FEV_1_), with a 15-min waiting period between two doses. Four puffs of albuterol were administered in the first dose and two (subjects < 16 years old) to four (subjects ≥ 16 years old) puffs were given in the second dose. Spirometry was performed fifteen minutes after each dose of albuterol to obtain post-FEV_1_ measurements and the higher of those was used to determine BDR. For CAMP, GACRS and HPR, spirometry was conducted following American Thoracic Society recommendations. The best FEV1 and FVC were selected for data analysis. After completing baseline spirometry, subjects were given 200 µg (2 puffs) of an albuterol metered-dose inhaler using a spacer, and spirometry was repeated after 15 min^11,18,29^. For all subjects, BDR was calculated as the percent change in post-FEV1 compared to pre-FEV_1_; that is BDR (ΔFEV_1_) = (post-FEV_1_ – pre-FEV_1_) / pre-FEV_1._

### Trans-omics for precision medicine (TOPMed) whole-genome sequencing (WGS) data

DNA samples for SAGE II, GALA II, SAPPHIRE, and GCPD-A subjects were sequenced as part of the Trans-Omics for Precision Medicine (TOPMed) WGS program^30^. WGS was conducted at the New York Genome Center and Northwest Genomics Center using a HiSeq X system (Illumina, San Diego, CA) with a mean depth of at least 30X (paired end, 150-bp reads). Full details of DNA sample handling, quality control, library construction, clustering and sequencing, read processing, and sequence data quality control are available in the TOPMed website (https://www.nhlbiwgs.org/). Briefly, demultiplexing of sequencing data was performed with bcl2fastq (Illumina, San Diego, CA, USA), sequencing data was aligned to human reference build 38 (GRCh38 with decoy) using BWA-MEM^31^, and data were further processed with the GATK best-practices pipeline^32^. Quality control procedures included marking of duplicate reads using Picard tools^33^, realignment around indels, and base quality recalibration. Single-sample genotypes were called using the GATK haplotype caller followed by joint genotyping of all subjects. The resulting multi-sample Variant Call Format (VCF) file was used for variant quality control score recalibration (VQSR). A 99.8% truth sensitivity trache level was used for SNPs and 99.0% for indel variants. SNP calls were used to check for sample contamination, and sample identity was confirmed by requiring > 99.5% concordance with SNP array (HumanCoreExome-24 array) genotypes. As part of the TOPMed program, BAM files were submitted to the Informatics Resource Center (IRC) at the University of Michigan and all samples passed TOPMed’s IRC quality control metric (mean genotype coverage > 30X; > 95% of genome covered at > 10X; and < 3% contamination). Genotype consistency between WGS data and previously published Axiom Genome-Wide LAT 1 array (Affymetrix, Santa Clara, CA) genotype data (dbGaP phs000920.v1.p1 and phs000921.v1.p1) was assessed using VCFtools^34^. Samples with percentage consistency three standard deviations below the mean were excluded. Downstream analyses were performed only on variants that passed quality control filters.

### Primary BDR GWAS

Genetic data for each racial/ethnic group was analyzed separately to avoid confounding due to major differences in population stratification. For subjects from the GALA II, SAGE II, and GCPD-A cohorts, association analyses were performed on the ENCORE server (https://encore.sph.umich.edu/) using TOPMed freeze 8 data (DP0 PASS) with SAIGE linear mixed model^35^ to account for relatedness and fine-scale population structure. The inverse-normalize-response option was enabled to address non-normality of BDR distributions. Age, sex, body mass index (BMI) category, sequencing batch, and the first four genetic principal components were included as covariates. For participants under 20 years old, standardized age- and sex-specific growth charts were used to calculate BMI percentiles and categorize BMI as follows: underweight (BMI percentile < 5th), normal (5th ≤ BMI percentile < 85th), overweight (85th ≤ BMI percentile < 95th) and obese (BMI percentile ≥ 95th). For participants aged 20 years and older, BMI categories were assigned as follows: underweight (BMI < 18), normal (18 ≤ BMI < 25), overweight (25 ≤ BMI < 30) and obese (BMI ≥ 30). Subjects were excluded if they had any missing covariate or BDR was not within 3 standard deviations of the mean. At the genotype level, variants with minor allele frequency (MAF) < 0.01 were excluded. For subjects from the SAPPHIRE cohort, GWAS was performed using the R package GENESIS^36^ with TOPMed freeze 7 data (DP10 PASS) based on the same model and covariates as used with GALA II, SAGE II, and SAPPHIRE. To evaluate variant-level associations in African Americans, a meta-analysis was performed with the SAGE II, SAPPHIRE, and GCPD-A results using METASOFT^37^ with the fixed effects model based on inverse-variance-weighted effect sizes. A trans-ethnic meta-analysis was also conducted across the three minority populations using the GWAS results of all cohorts.

### Replication analysis of BDR-associated variants

For CAMP, genotyping was performed at the Channing Division of Network Medicine using either the Illumina Quad 610 or Illumina 550 microarray chips (Illumina, Inc., San Diego, CA). For GACRS, genotyping was performed at the Channing Division of Network Medicine using the Illumina BeadStation 500G platform (Illumina Inc., San Diego CA). Genotype imputation was performed with the Michigan Imputation Server^38^ using the Haplotype Reference Consortium (HRC) r1.1 2016^39^ as the reference panel, and only SNPs with ≥ 0.05 and imputation quality r^2^ ≥ 0.3 were kept. For HPR, genotyping was conducted using the HumanOmni2.5 BeadChip platform (Illumina Inc., San Diego, CA) and imputation of non-genotyped variants was performed with IMPUTE2 using data from Phase I of the 1000 Genomes Project as the reference panel, as previously described^27^. Genetic associations were evaluated with linear regression models, adjusting for genetic principal components, age, sex, BMI category, and (if appropriate) study site.

### Region-based gene association tests using GWAS summary statistics

We performed region-based gene association tests with the snpsettest package (https://cran.r-project.org/package=snpsettest)^40^ in R 4.0.4 (R Core Team 2021) using gene annotations from the GENCODE project^41^. We considered genes in the following biotypes: protein-coding, immunoglobulin variable chain and T-cell receptor genes. For each gene, any variants within 20 kb of the 5’ and 3’ UTRs were selected for association tests, and the 1000 Genomes Project phase 3 African Americans and Admixed American panels^42^ were used to infer relationships among markers. A significant association was defined as having a Bonferroni-corrected *P* < 0.05.

### Predicted quantitative trait loci (QTL) analysis of GWAS data

We used the S-PrediXcan framework^43^ to correlate BDR to monocyte gene expression levels as predicted using data from the Multi-Ethnic Study of Atherosclerosis (MESA) cohort, which was optimized to predict gene expression within and across diverse populations^44^. For testing the association between the predicted gene expression levels of participants in each racial/ethnic group (i.e., African American, Mexican, Puerto Rican) and BDR, we used the MESA ALL model (https://doi.org/10.5281/zenodo.3610513) trained using pooled samples of African American, Hispanic, and European participants in the MESA cohort.

### Validation with multiomics data

To investigate functional target genes for association signals that lie distant to protein-coding genes, publicly available chromatin interaction data were explored using the 3D Genome browser^45^. Specifically, capture Hi-C data were utilized to identify long-range interactions that involved GWAS hits with promoters of distant gene candidates^46^. Transcriptomic analysis was carried out to investigate whether potential candidate genes identified via GWAS were differentially expressed by asthma-related conditions. Publicly available transcriptomic datasets related to asthma and asthma drug response were retrieved from the Gene Expression Omnibus (GEO)^47^ and analyzed with the RAVED pipeline^48^. Differential expression results comparing 1) patients with asthma versus healthy controls, or 2) asthma-related drug exposure versus vehicle control in vitro were obtained for three datasets. The Benjamini–Hochberg approach was used to adjust for multiple comparisons of genes/probes tested within each dataset and a q-value < 0.05 was considered significant. Single-tissue eQTL data from the Genotype-Tissue Expression (GTEx) v8 release^49^ were explored to identify GWAS hits that were also eQTL in lung, whole blood, and skeletal muscle of candidate genes.

## Results

Subject characteristics are provided in Table 1. A total of 1,136 Puerto Ricans, 656 Mexicans and 4,337 African Americans were included in the primary cohorts. The SAPPHIRE subjects were different from the other cohorts in that they were older and had a lower proportion of males. Principal components analysis of genotype data separated the study subjects according to racial/ethnic groups, demonstrating differences in genetic ancestry (Supplementary Fig. S1). The replication cohorts included 560 white, 967 Costa Rican, and 417 Puerto Rican subjects. White and Costa Rican patients had a lower proportion of obese subjects compared to the remainder of the cohorts.

**Table 1:** Descriptive characteristics of the study subjects.

	Primary Cohorts					Replication cohorts		
	GALA II		SAGE II	SAPPHIRE	GCPD-A	CAMP	GACRS	HPR
Racial/ethnic group	Puerto Rican	Mexican	African American	African American	African American	White	Costa Rican	Puerto Rican
N	1136	656	923	3127	287	560	967	417
Male, %	53.3	55.6	50.4	35.1	51.0	59.8	53.8	54.2
Age, median (IQR)	12.1 (4.9)	12.5 (5.4)	13.9 (6.1)	32.2 (25.5)	12.4 (6.6)	8.8 (3.5)	9.1 (2.9)	10.0 (4.0)
BDR, median (IQR)	11.2 (8.1)	7.1 (6.9)	8.5 (7.5)	6.1 (11.7)	5.4 (11.7)	8.0 (11.0)	4.2 (8.3)	4.0 (10.2)
**BMI category, %**								
Underweight	5.8	0.9	0.7	1.8	5.2	2.7	3.5	2.1
Normal	47.6	37.5	45.4	32.6	50.2	67.9	65.7	48.7
Overweight	16.2	19.7	19.9	19.5	11.8	15.5	14.7	17.5
Obese	30.4	41.9	34.0	46.2	32.8	13.9	16.0	31.7

IQR: interquartile range (3rd quantile—1st quantile).

**Figure 1:**
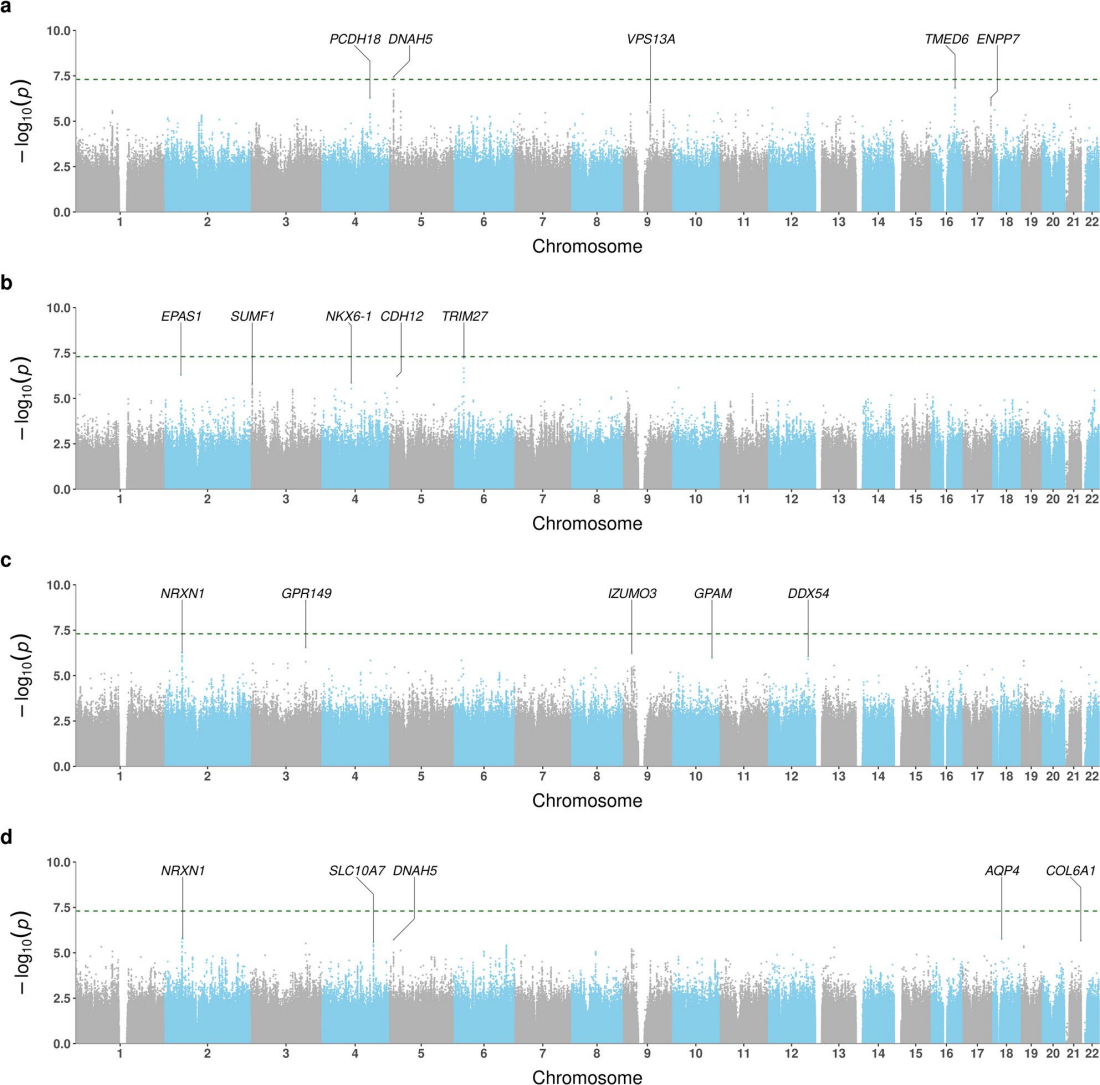
Manhattan plots of BDR GWAS results for (**a**) Puerto Ricans, (**b**) Mexicans, (**c**) African Americans, and (**d**) trans-ethnic meta-analysis. Top 5 loci of each result were annotated with the nearest protein-coding genes. The green horizontal dashed line indicates a genome-wide significance threshold of 5 × 10^–8^.

**Figure 2:**
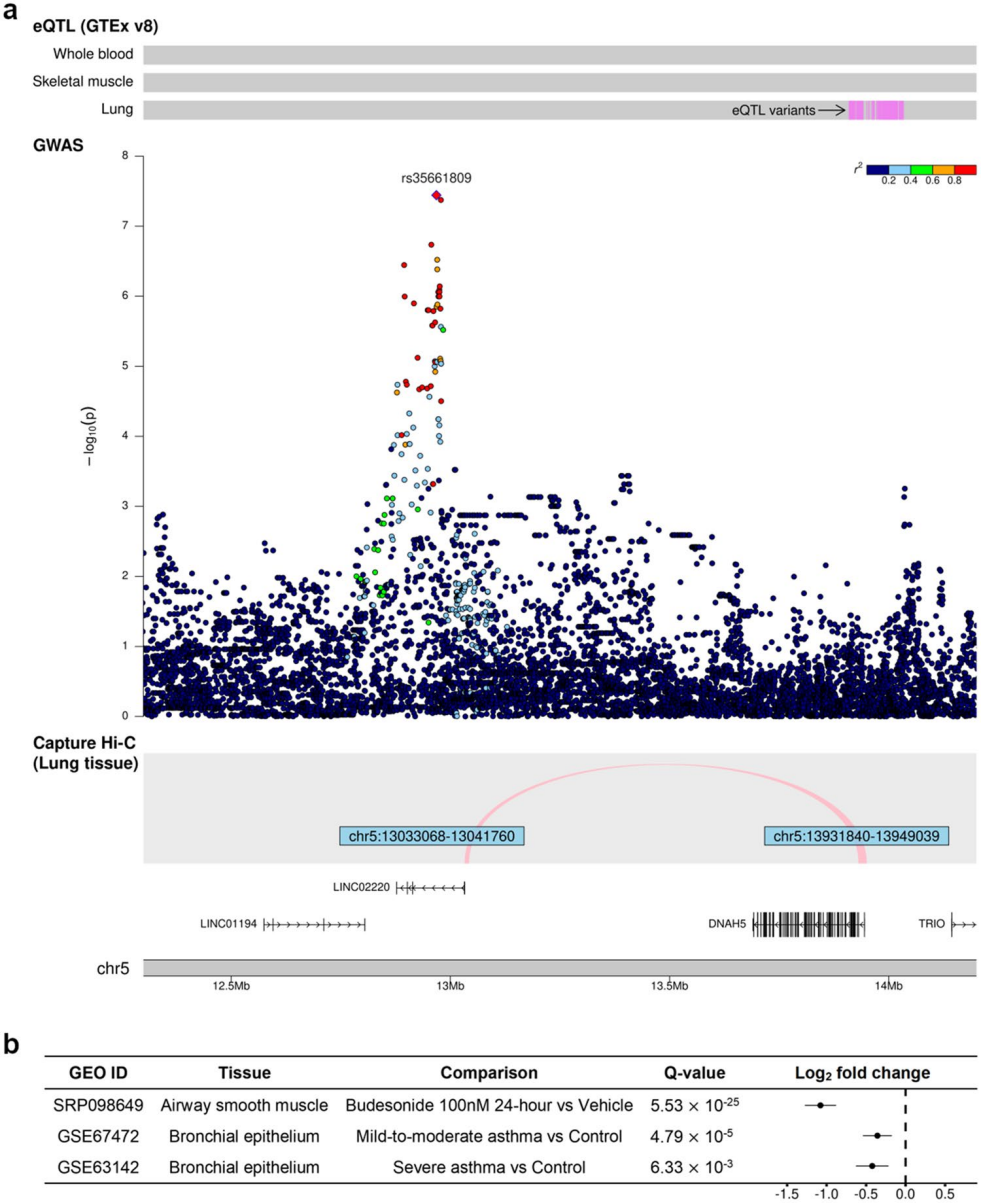
Evidence supporting *DNAH5* as a BDR-associated gene in Puerto Ricans. (**a**) eQTL (top) from the GTEx v8 release, regional associations in the GWAS (middle), and a chromatin interaction in lung tissue (bottom) from the Capture Hi-C data. (**b**) differential expression results for *DNAH5* using the GEO data.

The Manhattan plots for BDR GWAS are shown in Fig. 1. In Puerto Ricans, we found one genome-wide significant locus at 5p15.2 (Table 2). The lead variant, rs35661809 (*P* = 3.61 × 10^–8^), was located in the non-coding RNA gene *LINC02220* and was > 700 kb away to *DNHA5*, the nearest coding gene (Fig. 2A). Capture Hi-C data revealed a long-range interaction between this locus and the promoter of *DNAH5* in lung tissue, suggesting that *DNAH5* may be a functional target gene. The GTEx data demonstrated that several variants around the promoter region of *DNAH5* were eQTL in lung. According to transcriptomic data, *DNAH5* had decreased expression in bronchial epithelium of asthma patients versus controls and in airway smooth muscle treated with budesonide 100 nM for 24 h (Fig. 2B). The other highly ranked regions included loci at 16q22.1 (rs201865968, *P* = 1.51 × 10^–7^), 17q25.3 (rs4890030, *P* = 5.14 × 10^–7^), and 4q28.3 (rs2634863, *P* = 5.27 × 10^–7^).

**Table 2:** Top 5 loci and their lead variants in BDR GWAS.

	rsID	Position	RA	RAF	Beta (SE)	P value	Gene
**Puerto Rican**							
5p15.2	rs35661809	5:12,968,229	G	0.324	0.246 (0.045)	3.61 × 10^–8^	*DNAH5* (722 kb)
16q22.1	rs201865968	16:69,345,393	G	0.015	0.939 (0.179)	1.51 × 10^–7^	*TMED6*
17q25.3	rs4890030	17:79,750,299	A	0.684	−0.230 (0.046)	5.14 × 10^–7^	*ENPP7*
4q28.3	rs2634863	4:136,835,843	C	0.856	0.310 (0.062)	5.27 × 10^–7^	*PCDH18* (683 kb)
9q21.2	rs17063600	9:77,402,879	A	0.096	0.356 (0.073)	9.30 × 10^–7^	*VPS13A*
**Mexican**							
6p22.1	rs138319258	6:28,946,951	T	0.011	1.405 (0.259)	6.08 × 10^–8^	*TRIM27* (23 kb)
2p21	rs72797438	2:46,409,366	A	0.024	0.925 (0.184)	5.15 × 10^–7^	*EPAS1* (23 kb)
5p14.3	rs145081988	5:22,508,304	A	0.040	0.678 (0.136)	6.10 × 10^–7^	*CDH12*
4q21.23	rs78023565	4:84,177,276	T	0.039	0.706 (0.146)	1.42 × 10^–6^	*NKX6-1* (315 kb)
3p26.1	rs793940	3:4,372,905	T	0.226	0.321 (0.067)	1.60 × 10^–6^	*SUMF1*
**African American**							
3q25.2	rs16824202	3:154,570,767	A	0.346	−0.119 (0.023)	2.83 × 10^–7^	*GPR149* (140 kb)
9p21.3	rs7866009	9:25,043,077	G	0.223	−0.134 (0.027)	5.44 × 10^–7^	*IZUMO3* (497 kb)
2p16.3	rs28657436	2:49,568,516	T	0.187	0.140 (0.028)	5.58 × 10^–7^	*NRXN1* (350 kb)
12q24.13	rs140437863	12:113,168,186	A	0.033	−0.304 (0.062)	8.86 × 10^–7^	*DDX54*
10q25.2	rs116635459	10:112,234,664	T	0.027	−0.335 (0.069)	1.06 × 10^–6^	*GPAM* (193 kb)
**Trans-ethnic Meta-analysis**							
2p16.3	rs13007362	2:51,278,437	T	0.110	0.140 (0.029)	1.66 × 10^–6^	*NRXN1* (53 kb)
18q11.2	rs2239214	18:26,869,561	A	0.216	0.116 (0.024)	1.75 × 10^–6^	*AQP4* (38 kb)
5p15.2	rs1017451	5:12,974,996	T	0.150	0.114 (0.024)	1.93 × 10^–6^	*DNAH5* (715 kb)
21q22.3	rs9979315	21:46,015,014	A	0.340	0.095 (0.020)	2.19 × 10^–6^	*COL6A1* (10 kb)
4q31.22	rs34213717	4:146,524,351	A	0.246	0.097 (0.021)	2.67 × 10^–6^	*SLC10A7* (2 kb)

Genomic position is based on GRCh38. The nearest protein-coding gene was defined by kb distance with respect to the lead variant when it is not located within the gene. RA: risk allele; RAF: risk allele frequency; SE: standard error.

While no association achieved genome-wide significance in Mexicans and African Americans, several variants were associated with BDR at *P* < 1 × 10^–6^. Among Mexicans, a locus at 6p22.1 (rs138319258, *P* = 6.08 × 10^–8^) showed the strongest association with BDR, followed by loci at 2p21 (rs72797438, *P* = 5.15 × 10^–7^) and 5p14.3 (rs145081988, *P* = 6.10 × 10^–7^). Among African Americans, the top loci were found at 3q25.2 (rs16824202, *P* = 2.83 × 10^–7^), 9p21.3 (rs7866009, *P* = 5.44 × 10^–7^), and 2p16.3 (rs28657436, *P* = 5.58 × 10^–7^).

No loci reached genome-wide significance in the trans-ethnic meta-analysis. The lowest p values were observed in loci at 2p16.3 (rs13077362, *P* = 1.66 × 10^–6^), 18q11.2 (rs2239215, *P* = 1.75 × 10–6), and 5p15.2 (rs1017451, *P* = 1.93 × 10^–6^).

A total of 50 variants were selected for replication based on having a genome-wide significant association (i.e., 5p15.2 region found in Puerto Ricans) or *P* value of < 10^–5^ in the trans-ethnic meta-analysis (Supplementary Table S1). Several variants at 5p15.2, including the lead variant rs35661809 in Puerto Ricans, were nominally associated (1-tailed *P* < 0.05) with BDR in the GACRS cohort. None of the other top associations were replicated in the independent cohorts.

The region-based gene association tests showed no significant associations at the Bonferroni threshold (Supplementary Fig. S2). In addition, none of the predicted monocyte expression levels were associated with BDR, although *LIN7A* (*P* = 1.27 × 10^–5^) approached the Bonferroni significance level in Mexican patients with asthma (Supplementary Fig. S3).

Finally, we examined previously reported BDR-associated variants in/near *ADRB2*, *ADCY9*, *ARG1*, *COL22A1*, *CRHR2*, *GLCCI1*, *GSNOR*, *THRB*, and *SPATS2L*, but observed no significant associations in our study (Supplementary Table S2).

## Discussion

Our racial/ethnic population-specific GWAS identified one genome-wide significant locus at 5p15.2 in Puerto Ricans, for which the lead variant (rs35661809) was located in the non-coding RNA gene *LINC02220*. This region was previously identified as a BDR-associated locus in a study of GALA II and SAGE II subjects that used as outcome extreme responders to albuterol sampled from the tails of the BDR distribution, although that study did not provide evidence that linked the associated region to the gene other than physical proximity^4^. Overall, our results are largely consistent with this previous study, but by using a greater number of subjects and a continuous distribution of BDR to identify this association, we additionally found that 1) capture Hi-C data illustrated a long-range interaction with the promoter of *DNAH5* in lung tissue, suggesting its potential role in the transcription of *DNAH5*; and 2) several variants in the promoter region of *DNAH5* were significant eQTL in lung tissue consistent with the Hi-C finding. A recent GWAS based on UK Biobank data identified an association between an intronic *DNAH5* variant and the lung function measure FEV_1_/FVC ratio (FVC: Forced vital capacity) among adults^50^, and a GWAS from the COPDGene Study reported suggestive associations between *DNAH5* variants and post-bronchodilator FEV_1_/FVC among smokers of European and African ancestry that were more pronounced (P value < 5 × 10^–6^) when restricting the analysis to those with COPD^51^.

*DNAH5* encodes a dynein protein, which functions as a force-generating protein of respiratory cilia and plays an important role in cilia mobility, especially in respiratory epithelial cells, where ciliary motility is indispensable to mucus transport and airway clearance^52,53^. Mutations in *DNAH5* have been linked to primary ciliary dyskinesia (PCD), which is characterized by marked airway dysfunction^54,55^. Our transcriptomic results provide evidence of the relevance of *DNAH5* to asthma, given that its gene expression levels were decreased in bronchial epithelium of patients with asthma versus controls and in airway smooth muscle cells after budesonide exposure. Although our replication results found that rs35661809 and several *LINC02220* intronic variants were nominally associated with BDR with a consistent direction of effect in the GACRS cohort, the replication was not observed in CAMP or HPR. Taken these together, our findings suggest that *DNAH5* contributes to variability of BDR, but a more precise mechanistic link between the associated locus and *DNAH5* with BDR has yet to be elucidated.

Although we did not observe genome-wide significant associations in Mexicans and African Americans, the top loci may be of interest for future studies. In Mexicans, rs138319258 near *TRIM27* at 6p22.1 had the lowest *P* value with BDR. The tripartite motif containing protein 27 (TRIM27) functions as an E3 ligase to ubiquitinate and inhibit Phosphatidlyinositol-3-kinase C2 beta (PI3KC2β), which plays an important role in the IgE receptor (FcεRI) and mast cell activation^56^. In allergic asthma, mast cells are activated mainly through IgE-mediated cross-linking of FcεRI with allergens^57^. The role of TRIM27 in mast cell regulation indicates the potential relationship between genetic variations in *TRIM27* and asthma. Along the same lines, previous GWAS have identified an association between a locus near *TRIM27* and asthma^58,59^, and previous studies have shown that IgE levels are associated with BDR^3,60^. In African Americans, the most strongly associated variant was rs16824202 adjacent to *GPR149* at 3q25.2. Variants in the *GPR149* gene, which encodes a seven-transmembrane G protein-coupled receptor (GPCR) class A family member, have been associated with coronary artery disease, HDL cholesterol level, and apolipoprotein A1 level^61,62^.

We did not identify significant associations in the trans-ethnic meta-analysis. The top variant, rs13000632 at 2p16.3, was located near the *NRXN1* gene that has been associated with smoking behaviors across diverse populations^63–65^. Given that smokers with asthma have worse outcomes compared to non-smokers with asthma^66,67^, there may be an interaction between polymorphisms in smoking-related genes and BDR. Another top independent locus was found at at 18q11.2. near *AQP4*, a gene whose expression was up-regulated in bronchial epithelial cells from patients with asthma^68^, and whose altered regulation was also observed in allergen and IL-13-induced mouse models of asthma^69^. Future validation studies are needed to substantiate the functional relationships between *NRXN1* and *AQP4* with BDR.

Our region-based gene association tests that evaluated the combined effects of variants within prespecified gene boundaries did not find statistically significant associations. To overcome the limitations inherent in assigning a potential functional link to a gene based on proximity of variants alone, we additionally performed predicted QTL analysis to investigate whether a set of variants regulated the expression of a specific gene that was associated with BDR. Due to the paucity of gene expression data for people of non-European ancestry, we were only able to examine associations with monocyte gene expression levels predicted from the MESA study^44^. Expanding gene expression data to include more diverse people and more asthma-related tissues (e.g., lung, airway smooth muscle, bronchial epithelium) would be of interest to elucidate tissue-specific BDR-related pathways.

Genetic variation of the β_2_-adrenergic receptor (*ADRB2*) gene, which encodes the direct target of SABAs, has been of particular interest in BDR studies, but associations between *ADRB2* variants and BDR have not been consistently observed^70^. Our results did not identify BDR-associated variants in *ADRB2* or other previously identified candidate genes, which may be in part explained by differences in study design and the fact that results from candidate gene studies have been difficult to replicate^71^. Several asthma GWAS have been identified strong and consistently replicated genetic associations^72^, including novel loci in a recent study focused on moderate-to-severe asthma^73^, but thus far, the asthma susceptibility loci identified have not been linked to BDR pathways. The top BDR-associated loci identified in this study are also not among the previously identified asthma-associated variants.

Our study has several limitations. First, the sample size of the current study was not sufficient to detect modest effect sizes of single-variant associations that might show marginal levels of significance, although it is one of the largest pharmacogenetic studies of minority populations using whole-genome sequencing data to date. Second, there was heterogeneity in subject characteristics across our primary cohorts. Notably, subjects from SAPPHIRE were older than the other cohorts, and therefore, may have different clinical characteristics that influenced BDR. Finally, we were not able to identify additional asthma cohorts of Mexican and African American populations to replicate our primary findings. Future studies that consider population-specific differences in BDR heterogeneity, are better able to account for differences in patterns of linkage disequilibrium among populations of different genetic ancestry, and are of larger sample sizes, may uncover more robust BDR associations and find overlap among genetic risk factors of asthma and BDR.

In summary, by leveraging multiomics data, including capture Hi-C, gene expression, and eQTL, we found that *DNAH5* may influence BDR, and its genetic variation may contribute to differences in asthma outcomes among Puerto Ricans. Future mechanistic studies should be pursued to identify how *DNAH5* influences BDR.

## Data availability

The datasets used and/or analyzed during the current study are available from the PIs of the respective studies upon reasonable request. The TOPMed WGS data analyzed during the current study are available in dbGaP with the following accession numbers: phs000920 (GALA II), phs000921 (SAGE II), phs001467 (SAPPHIRE), and phs001661 (GCPD-A). MESA PrediXcan prediction model: https://doi.org/10.5281/zenodo.3610513. 3D Genome Browser Capture Hi-C: http://3dgenome.fsm.northwestern.edu. Gene Expression Omnibus (GEO) repository: https://www.ncbi.nlm.nih.gov/geo (GED ID: SRP098649; GSE67472; GSE63142) Genotype-Tissue Expression (GTEx) v8 release: https://www.gtexportal.org/home/datasets.

## Supplementary Information

Supplementary Information 1.

Supplementary Information 2.

## Abbreviations

BDR: Bronchodilator response
SABAs: Short-acting β_2_-agonists
PKA: CAMP-dependent protein kinase A
FEV_1_: Forced expiratory volume in 1 s
FVC: Forced vital capacity
GWAS: Genome-wide association study
QTL: Quantitative trait loci
TOPMed: Trans-Omics for Precision Medicine
GEO: Gene Expression Omnibus
GTEx: Genotype-Tissue Expression
SAGE II: Study of African Americans, Asthma, Genes, and Environments
GALA II: Genes-environments and Admixture in Latino Americans
SAPPHIRE: Study of Asthma Phenotypes and Pharmacogenomic Interactions by Race-Ethnicity
GCPD-A: Genetic Causes of Complex Pediatric Disorders—Asthma
CAMP: Childhood Asthma Management Program
GACRS: The Genetics of Asthma in Costa Rica Study
HPR: Hartford-Puerto Rico
ATGC: Asthma Translational Genomic Collaboration
MESA: Multi-Ethnic Study of Atherosclerosis
