# Supplementary material for: Genome-wide association study in minority children with asthma implicates DNAH5 in bronchodilator responsiveness
Source: Sci Rep. 2022 Jul 22;12:12514. doi: 10.1038/s41598-022-16488-6 (PMC9307508; doi:10.1038/s41598-022-16488-6)
Supplement: Supplementary file 1 — Supplementary Information 1. [file 41598_2022_16488_MOESM1_ESM.pptx]

## Slide 1
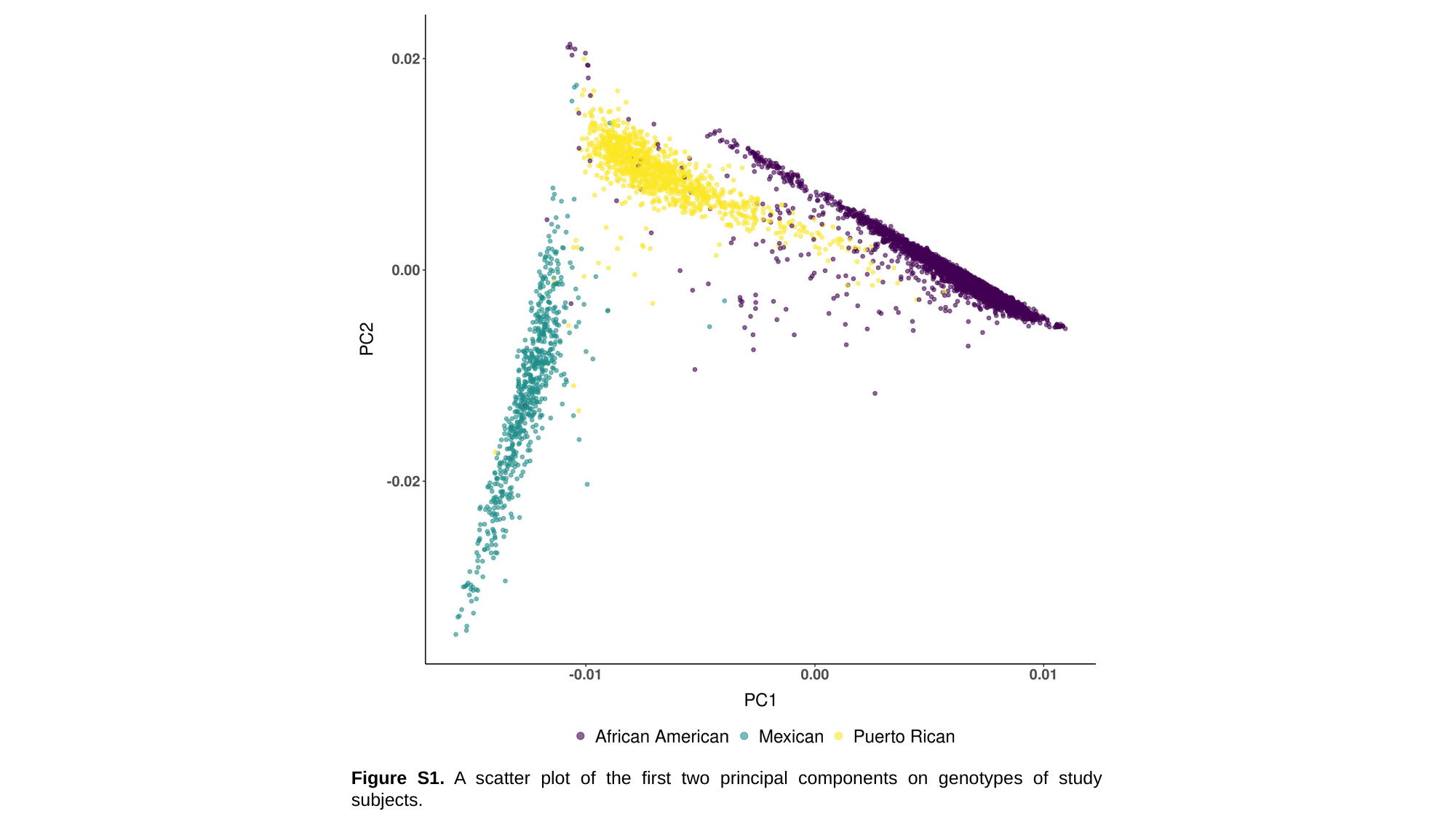

Figure S1. A scatter plot of the first two principal components on genotypes of study subjects.

## Slide 2
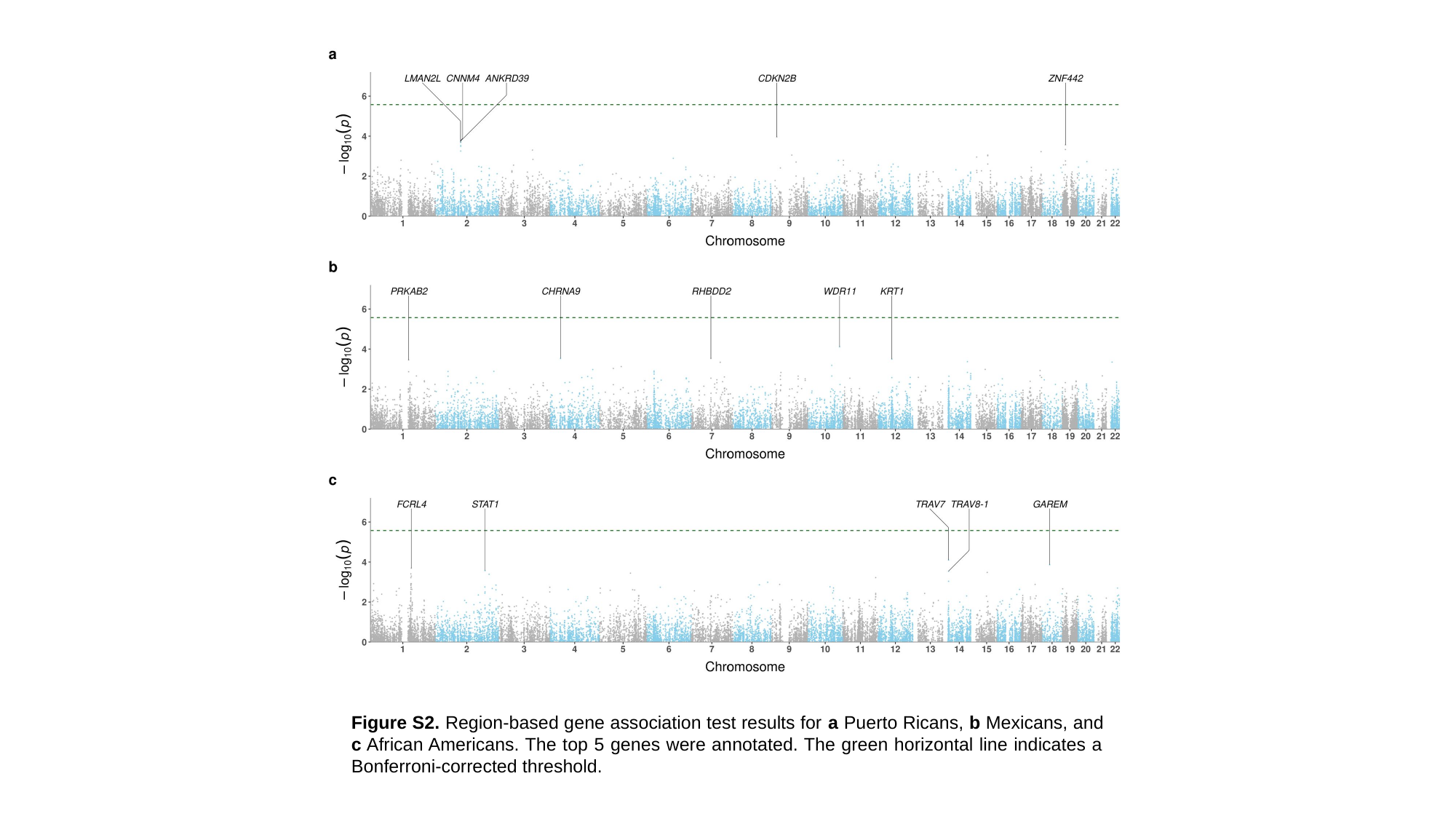

Figure S2. Region-based gene association test results for a Puerto Ricans, b Mexicans, and c African Americans. The top 5 genes were annotated. The green horizontal line indicates a Bonferroni-corrected threshold.

## Slide 3
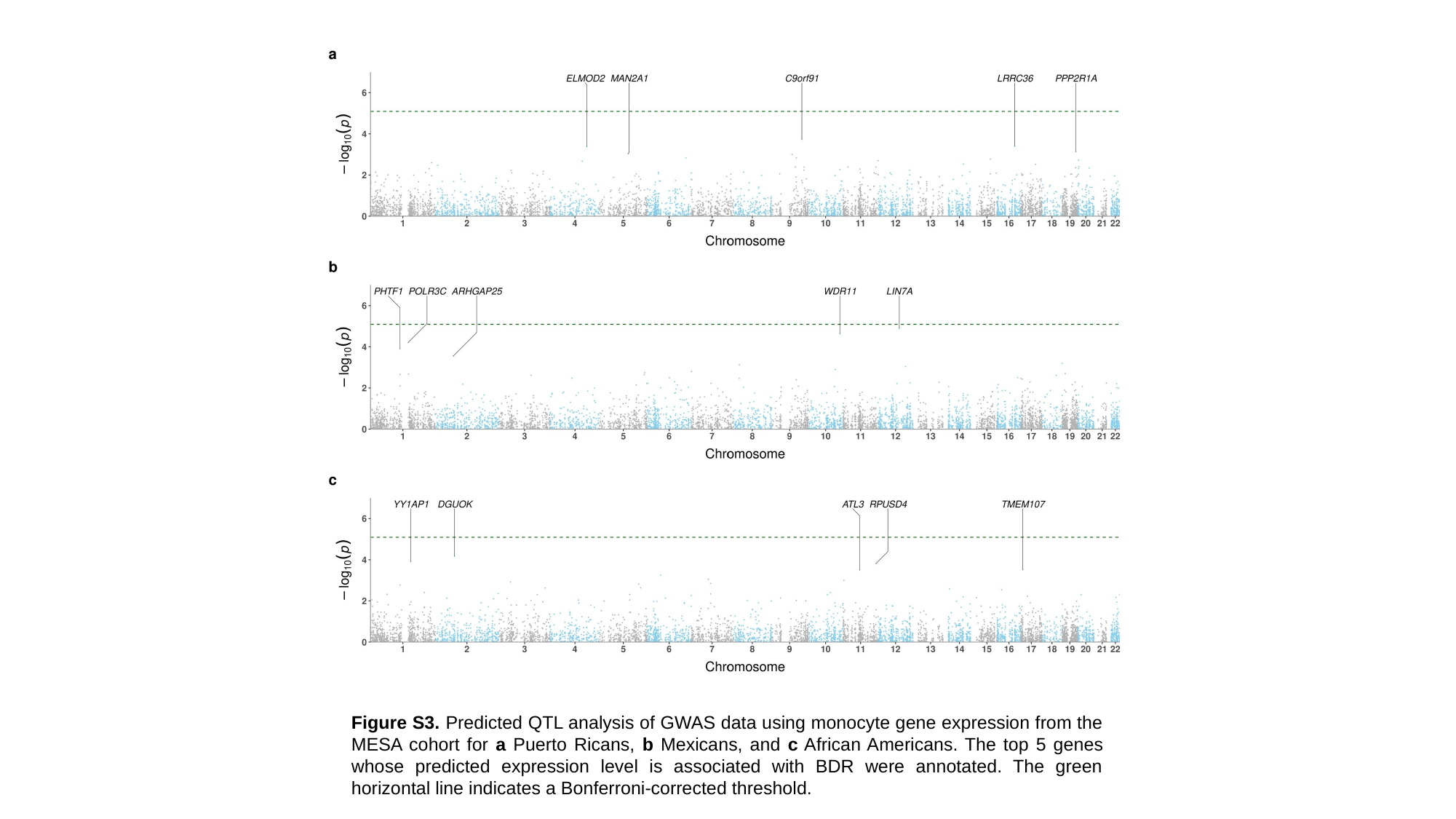

Figure S3. Predicted QTL analysis of GWAS data using monocyte gene expression from the MESA cohort for a Puerto Ricans, b Mexicans, and c African Americans. The top 5 genes whose predicted expression level is associated with BDR were annotated. The green horizontal line indicates a Bonferroni-corrected threshold.
