# Supplementary material for: Genome-wide association study in minority children with asthma implicates DNAH5 in bronchodilator responsiveness
Source: Sci Rep. 2022 Jul 22;12:12514. doi: 10.1038/s41598-022-16488-6 (PMC9307508; doi:10.1038/s41598-022-16488-6)
Supplement: Supplementary file 2 — Supplementary Information 2. [file 41598_2022_16488_MOESM2_ESM.docx]

**Table S1.** Association of top BDR GWAS variants in replication cohorts.

|  |  |  |  | Current Study | | Replication cohorts | | | | | |
| --- | --- | --- | --- | --- | --- | --- | --- | --- | --- | --- | --- |
|  |  |  |  | Trans-ethnic Meta-analysis | | CAMP | | Costa Rica | | Hartford-Puerto Rico | |
| **rsID^*^** | **Position** | **RA** | **Gene^†^** | **Beta** | **P^‡^** | **Beta** | **P**^¶^ | **Beta** | **P**^¶^ | **Beta** | **P**^¶^ |
| rs58539681 | 1:71818428 | A | *NEGR1* | 0.108 | 4.71 x 10^-6^ | 0.083 | 0.243 | 0.007 | 0.456 | - | - |
| rs4381210 | 1:102781741 | T | *COL11A1* | 0.088 | 8.34 x 10^-6^ | -0.168 | 0.997 | 0.025 | 0.307 | 0.050 | 0.248 |
| rs7592006 | 2:49566345 | C | *NRXN1* | 0.113 | 9.09 x 10^-6^ | -0.067 | 0.661 | -0.042 | 0.640 | 0.040 | 0.376 |
| rs7578534 | 2:49566654 | C | *NRXN1* | 0.118 | 4.63 x 10^-6^ | -0.067 | 0.661 | -0.042 | 0.640 | 0.040 | 0.376 |
| rs28657436 | 2:49568516 | T | *NRXN1* | 0.124 | 1.69 x 10^-6^ | -0.067 | 0.661 | -0.042 | 0.640 | 0.041 | 0.375 |
| rs7595953 | 2:49569551 | G | *NRXN1* | 0.118 | 5.12 x 10^-6^ | -0.067 | 0.661 | -0.042 | 0.640 | 0.039 | 0.380 |
| rs10189258 | 2:49570454 | T | *NRXN1* | 0.119 | 4.06 x 10^-6^ | -0.067 | 0.661 | -0.042 | 0.640 | 0.046 | 0.358 |
| rs2133329 | 2:49575828 | T | *NRXN1* | 0.122 | 2.54 x 10^-6^ | -0.067 | 0.661 | -0.042 | 0.640 | 0.049 | 0.350 |
| rs13007362 | 2:51278437 | T | *NRXN1* | 0.14 | 1.66 x 10^-6^ | -0.133 | 0.907 | 0.107 | 0.068 | -0.03 | 0.602 |
| rs56036910 | 2:58943751 | A | *FANCL* | -0.09 | 9.95 x 10^-6^ | 0.004 | 0.525 | -0.068 | 0.090 | -0.08 | 0.152 |
| rs16824202 | 3:154570767 | A | *GPR149* | -0.102 | 3.08 x 10^-6^ | 0.058 | 0.533 | -0.083 | 0.335 | -0.078 | 0.262 |
| rs13152024 | 4:146508164 | A | *SLC10A7* | 0.094 | 4.28 x 10^-6^ | -0.021 | 0.621 | -0.037 | 0.784 | -0.14 | 0.956 |
| rs41398848 | 4:146522385 | T | *SLC10A7* | 0.103 | 4.50 x 10^-6^ | -0.02 | 0.617 | -0.048 | 0.845 | -0.084 | 0.853 |
| rs62326870 | 4:146522522 | G | *SLC10A7* | 0.096 | 3.56 x 10^-6^ | -0.02 | 0.617 | -0.05 | 0.852 | -0.064 | 0.791 |
| rs34213717 | 4:146524351 | A | *SLC10A7* | 0.097 | 2.67 x 10^-6^ | -0.027 | 0.658 | -0.056 | 0.884 | -0.063 | 0.788 |
| rs2357083 | 4:146525218 | A | *SLC10A7* | 0.104 | 7.04 x 10^-6^ | -0.076 | 0.840 | -0.022 | 0.682 | -0.078 | 0.824 |
| rs34845041 | 5:12948257 | T | *DNAH5* | 0.108 | 9.61 x 10^-6^ | -0.015 | 0.596 | 0.072 | 0.059 | 0.101 | 0.110 |
| rs62347395 | 5:12950320 | G | *DNAH5* | 0.108 | 9.63 x 10^-6^ | -0.015 | 0.593 | 0.072 | 0.059 | 0.099 | 0.113 |
| rs17833938 | 5:12961433 | A | *DNAH5* | 0.108 | 9.50x 10^-6^ | -0.012 | 0.571 | 0.076 | 0.049 | 0.091 | 0.136 |
| rs35661809 | 5:12968229 | G | *DNAH5* | 0.068 | 1.00 x 10^-3^ | -0.009 | 0.557 | 0.087 | 0.031 | - | - |
| rs35207506 | 5:12970021 | C | *DNAH5* | -0.05 | 7.95 x 10^-3^ | -0.002 | 0.487 | -0.082 | 0.038 | -0.061 | 0.205 |
| rs62349261 | 5:12970158 | G | *DNAH5* | -0.049 | 9.52 x 10^-3^ | 0.001 | 0.507 | -0.084 | 0.034 | -0.061 | 0.205 |
| rs17237443 | 5:12972524 | C | *DNAH5* | 0.109 | 1.84 x 10^-5^ | -0.002 | 0.510 | 0.073 | 0.058 | 0.107 | 0.097 |
| rs1017451 | 5:12974996 | T | *DNAH5* | 0.114 | 1.93 x 10^-6^ | -0.005 | 0.534 | 0.073 | 0.058 | 0.078 | 0.168 |
| rs1017453 | 5:12975153 | C | *DNAH5* | 0.095 | 3.29 x 10^-6^ | -0.012 | 0.575 | 0.073 | 0.058 | 0.089 | 0.136 |
| rs17237639 | 5:12975822 | G | *DNAH5* | 0.095 | 2.62 x 10^-5^ | -0.016 | 0.598 | 0.073 | 0.058 | 0.099 | 0.108 |
| rs17834628 | 5:12978454 | A | *DNAH5* | 0.073 | 0.12 x 10^-3^ | -0.02 | 0.623 | 0.078 | 0.046 | 0.093 | 0.124 |
| rs142458423 | 5:32449683 | T | *ZFR* | 0.264 | 7.47 x 10^-6^ | -0.09 | 0.759 | 0.006 | 0.477 | -0.164 | 0.811 |
| rs1590547 | 6:85097378 | T | *TBX18* | -0.108 | 8.32 x 10^-6^ | 0.054 | 0.677 | -0.019 | 0.394 | -0.072 | 0.267 |
| rs6910197 | 6:85104947 | A | *TBX18* | -0.108 | 9.27 x 10^-6^ | 0.054 | 0.677 | -0.019 | 0.394 | -0.073 | 0.262 |
| rs16875673 | 6:85109156 | C | *NT5E* | -0.108 | 8.64 x 10^-6^ | 0.054 | 0.677 | -0.019 | 0.394 | -0.073 | 0.262 |
| rs77130891 | 6:147998017 | T | *SAMD5* | -0.293 | 6.35 x 10^-6^ | 0.249 | 0.992 | 0.129 | 0.851 | 0.356 | 0.966 |
| rs11155537 | 6:147999920 | A | *SAMD5* | -0.291 | 7.78 x 10^-6^ | 0.251 | 0.993 | 0.129 | 0.851 | 0.356 | 0.966 |
| rs11155539 | 6:148001345 | T | *SAMD5* | -0.288 | 8.60 x 10^-6^ | 0.251 | 0.993 | 0.129 | 0.851 | 0.356 | 0.966 |
| rs12210121 | 6:148003352 | T | *SAMD5* | -0.289 | 7.38 x 10^-6^ | 0.257 | 0.994 | 0.129 | 0.851 | 0.356 | 0.966 |
| rs76997732 | 6:148018156 | C | *SASH1* | -0.297 | 4.78 x 10^-6^ | 0.270 | 0.996 | 0.050 | 0.663 | 0.356 | 0.966 |
| rs79699339 | 6:148022140 | T | *SASH1* | -0.297 | 4.63 x 10^-6^ | 0.282 | 0.997 | 0.050 | 0.663 | 0.356 | 0.966 |
| rs112514985 | 6:148057288 | A | *SASH1* | -0.294 | 6.15 x 10^-6^ | 0.273 | 0.997 | 0.050 | 0.663 | - | - |
| rs10957451 | 8:68801378 | T | *C8orf34* | 0.092 | 9.38 x 10^-6^ | -0.003 | 0.514 | -0.004 | 0.530 | -0.005 | 0.520 |
| rs10957452 | 8:68803146 | C | *C8orf34* | 0.092 | 8.81 x 10^-6^ | -0.003 | 0.514 | -0.004 | 0.530 | -0.005 | 0.520 |
| rs16935087 | 8:68803651 | T | *C8orf34* | 0.093 | 8.76 x 10^-6^ | -0.003 | 0.514 | -0.004 | 0.530 | -0.021 | 0.587 |
| rs7847175 | 9:25042727 | G | *IZUMO3* | -0.11 | 8.05 x 10^-6^ | -0.149 | 0.193 | -0.081 | 0.222 | 0.088 | 0.772 |
| rs7866009 | 9:25043077 | G | *IZUMO3* | -0.11 | 7.68 x 10^-6^ | -0.149 | 0.193 | -0.081 | 0.222 | 0.088 | 0.772 |
| rs73473766 | 9:25047394 | C | *IZUMO3* | -0.128 | 6.26 x 10^-6^ | 0.067 | 0.589 | -0.055 | 0.366 | 0.064 | 0.647 |
| rs7048804 | 9:25049242 | G | *IZUMO3* | -0.125 | 8.36 x 10^-6^ | 0.019 | 0.527 | -0.055 | 0.366 | 0.134 | 0.793 |
| rs76247099 | 13:52963506 | T | *OLFM4* | -0.171 | 5.09 x 10^-6^ | -0.242 | 0.137 | -0.073 | 0.341 | -0.160 | 0.173 |
| rs2339214 | 18:26869561 | A | *AQP4* | 0.116 | 1.75 x 10^-6^ | 0.054 | 0.173 | 0.012 | 0.395 | - | - |
| rs8108747 | 19:8603237 | T | *ADAMTS10* | -0.089 | 4.23 x 10^-6^ | 0.046 | 0.776 | 0.016 | 0.622 | 0.009 | 0.542 |
| rs4804316 | 19:8604551 | G | *ADAMTS10* | -0.088 | 5.11 x 10^-6^ | 0.046 | 0.779 | 0.016 | 0.621 | -0.001 | 0.497 |
| rs9979315 | 21:46015014 | A | *COL6A1* | 0.095 | 2.19 x 10^-6^ | 0.111 | 0.039 | 0.011 | 0.413 | 0.038 | 0.319 |

RA: risk allele

^*^ variants mapped to the genome-wide significant locus at 5p15.2 in the GWAS of Puerto Ricans or P value < 10^-5^ in the trans-ethnic meta-analysis

^†^ the nearest protein-coding gene

^‡^ P value is based on two-sided test

^¶^ P value is based on one-sided test

**Table S2.** Association of previously reported BDR-related variants in the current study.

|  |  |  |  |  |  |  | Current Study | | | | | | | |
| --- | --- | --- | --- | --- | --- | --- | --- | --- | --- | --- | --- | --- | --- | --- |
|  |  |  |  |  |  |  | Puerto Rican | | Mexican | | African American | | Trans-ethnic | |
| **PMID** | **Population** | **Gene** | **rsID** | **RA** | **Direction** | **Consequence^*^** | **Beta** | **P^#^** | **Beta** | **P** | **Beta** | **P** | **Beta** | **P** |
| 16931635 | African American | *ADRB2* | rs1042714 | C | + | stop-gained | -0.024 | 0.687 | 0.076 | 0.155 | - | - | - | - |
| 15557128 | Puerto Rican | *ADRB2* | rs1042713 | A | - | missense | 0.003 | 0.523 | -0.023 | 0.340 | -0.003 | 0.442 | -0.004 | 0.406 |
| 21545619 | Korean | *ADCY9* | rs2230739 | C | + | missense | 0.081 | 0.048 | -0.031 | 0.670 | 0.038 | 0.110 | 0.041 | 0.049 |
| 18617639 | European | *ARG1* | rs2781659 | G | - | none | -0.059 | 0.082 | -0.008 | 0.440 | - | - | - | - |
| 23508266 | European | *COL22A1* | rs6988229 | T | + | intron | 0.053 | 0.138 | -0.013 | 0.573 | - | - | - | - |
| 18408560 | European | *CRHR2* | rs7793837 | T | - | intron | -0.021 | 0.322 | -0.090 | 0.071 | - | - | - | - |
| 21991891 | European | *GLCCI1* | rs37972 | C | + | 2KB upstream | -0.060 | 0.904 | 0.012 | 0.414 | - | - | - | - |
| 19514054 | African American | *GSNOR* | rs1154400 | T | + | 2KB upstream | -0.044 | 0.830 | 0.011 | 0.443 | - | - | - | - |
| 23508266 | European | *THRB* | rs892940 | G | + | 2KB upstream | 0.030 | 0.250 | 0.012 | 0.418 | - | - | - | - |
| 22792082 | European | *SPATS2L* | rs295137 | T | + | none | -0.024 | 0.709 | -0.059 | 0.821 | 0.030 | 0.090 | 0.012 | 0.263 |

RA: risk allele; Direction: direction of effect

* based on dbSNP report (https://www.ncbi.nlm.nih.gov/snp)

^#^ based on one-sided test
